# Draft genome sequence of *Pantoea stewartii* subsp. *indologenes* strain UIA 44 isolated from discolored rice grain in a paddy field of Penang, Malaysia

**DOI:** 10.1128/mra.00410-26

**Published:** 2026-07-10

**Authors:** Sharifah Siti Maryam Syd Abdul Rahman, Suhaila Mohd. Omar, Nurul Hidayah Samsulrizal, Mohd Faizal Abu Bakar, Suhaila Sulaiman, Mohd Syahmi Salleh, Nur Sabrina Ahmad Azmi

**Affiliations:** 1 Department of Plant Science, Kulliyyah of Science, International Islamic University Malaysia (Kuantan Campus) 61774, Kuantan, Pahang, Malaysia; 2 Department of Biotechnology, Kulliyyah of Science, International Islamic University Malaysia (Kuantan Campus) 61774, Kuantan, Pahang, Malaysia; 3 Research Unit for Bioinformatic & Computational Biology (RUBIC), Kulliyyah of Science, International Islamic University Malaysia (Kuantan Campus) 61774, Kuantan, Pahang, Malaysia; 4 Advanced Genomics and Bioinformatics, Malaysia Genome and Vaccine Institute, National Institutes of Biotechnology Malaysia 455448, Kajang, Selangor, Malaysia; 5 FGV R&D Sdn. Bhd., FGV Innovation Center (Biotechnology) 473466 , Seremban, Negeri Sembilan, Malaysia; Loyola University Chicago, Chicago, Illinois, USA

**Keywords:** rice, *Pantoea stewartii *subsp. *indologenes*, whole-genome sequencing, pathogen, grain discoloration

## Abstract

*Pantoea stewartii* subsp. *indologenes* strain UIA 44 was isolated from discolored rice grains collected from a paddy field in Permatang Tinggi, Bakar Bata, Penang, Malaysia. This study presents the results from a whole-genome sequencing project to support further studies on its potential role as a rice-associated phytopathogen.

## ANNOUNCEMENT

*Pantoea stewartii* subsp. *indologenes* is a gram-negative bacterium within the family *Enterobacteriaceae*. Although genetically distinct from *P. stewartii* subsp. *stewartii*, the causative agent of Stewart’s wilt in maize (1)*,* recent findings indicated that *P. stewartii* subsp. *indologenes* is a potential pathogen of Poaceae species (2). It has been isolated from diseased tissues of grasses and cereals, exhibiting symptoms such as leaf necrosis, vascular discoloration, and wilting (3). More recently, it has also been linked to rice grain discoloration (4, 5).

*Pantoea stewartii* subsp. *indologenes* strain UIA 44 was isolated aseptically from naturally infected, symptomatic panicles exhibiting dark brown or black spots, grain discoloration, and hollow, lightweight structures. The samples were collected from a 0.6-ha paddy field in Permatang Tinggi, Bakar Bata, Penang, Malaysia (5.5655°N, 100.4351°E), and were transported to the laboratory in sterile polyethylene bags. Infected grains were surface-sterilized using 10% sodium hypochlorite for 1 min and 70% ethanol for 30 s, followed by three 5-min rinses in sterile distilled water. Surface-sterilized grains were placed directly without mechanical disruption onto King’s B agar and incubated at 37°C for 18–20 h. Single yellow colonies were then subcultured to ensure purity.

Genomic DNA was extracted from pure culture grown in Luria-Bertani broth at 37°C, 180 rpm for 18–20 h using the Lucigen MasterPure Complete DNA and RNA Purification Kit (Biosearch Technologies, LGC, North America), according to the manufacturer’s protocol. Identification was confirmed via PCR amplification and bidirectional Sanger sequencing (Apical Scientific Sdn. Bhd., Malaysia) of the 16S rRNA gene using the universal primers 27F (5′-AGAGTTTGATCCTGGCTCAG-3′) and 1492R (5′-GGTTACCTTGTTACGACTT-3′) (6, 7). Sequence analysis revealed 99.8% nucleotide identity with the type strain *P. stewartii* subsp. *indologenes* strain CIP 104006 (NR_104928.1).

A paired-end sequencing library was prepared using the NEBNext Ultra DNA Library Prep Kit (New England BioLabs, USA) and sequenced on the Illumina NovaSeq 6000 platform (Illumina, San Diego, CA, USA) with 150-bp paired-end reads at Apical Scientific Sdn Bhd (Selangor, Malaysia). Adapter trimming and quality filtering were carried out using BBDuk (BBMap v35.x) (8). *De novo* assembly was performed using VelvetOptimiser v2.2.5 (9), followed by scaffolding with SSPACE Standard v3.0 (10) and refinement with RagTag v1.1.0 (11). Genome completeness was evaluated using Benchmarking Universal Single-Copy Orthologs v5.2.2 (12) with the enterobacterales_odb10 data set. Default parameters were applied in all applications.

A total of 7,021,406 reads (1,053,210,900 bp) were generated, providing approximately 226× genome coverage. The final assembly consists of two scaffolds: a putative chromosome-level scaffold (4,400,953 bp) with 0.05% Ns and a plasmid scaffold (251,860 bp), totaling 4,652,813 bp, an *N*_50_ value of 4.6 Mbp, and a G + C content of 53.95%. BLASTn indicates the putative chromosome is closely related (99%) with strain CIP 104006 (NR_104928.1), while the plasmid is highly similar (99%) with *P. stewartii* strain ZJ-FGZX1 (CP049115.1). The genome completeness score is 99.4%. Overall, this high-quality genome sequence provides a foundation for future investigations on its potential role as a rice-associated pathogen.

## Data Availability

The whole-genome shotgun sequence of *P. stewartii* subsp. *indologenes* UIA 44 has been deposited in the National Center for Biotechnology Information (NCBI) GenBank under accession number JBSQDR000000000. The version described in this paper is the first version, JBSQDR000000000.1. The BioProject designation is PRJNA1264688, and the BioSample accession number is SAMN48576735. The raw sequencing paired-end reads have been deposited in the NCBI Sequence Read Archive with accession number SRR37670928.
